# A Sound Processor for Cochlear Implant Using a Simple Dual Path Nonlinear Model of Basilar Membrane

**DOI:** 10.1155/2013/153039

**Published:** 2013-04-17

**Authors:** Kyung Hwan Kim, Sung Jin Choi, Jin Ho Kim

**Affiliations:** ^1^Department of Biomedical Engineering, College of Health Science, Yonsei University, 234 Maeji-ri, Heungup-myun, Wonju, Kangwon-do 220-710, Republic of Korea; ^2^School of Electrical Engineering, Seoul National University, Shillim-dong, Kwanak-gu, Building 301, Seoul 151-742, Republic of Korea

## Abstract

We propose a new active nonlinear model of the frequency response of the basilar membrane in biological cochlea called the simple dual path nonlinear (SDPN) model and a novel sound processing strategy for cochlear implants (CIs) based upon this model. The SDPN model was developed to utilize the advantages of the level-dependent frequency response characteristics of the basilar membrane for robust formant representation under noisy conditions. In comparison to the dual resonance nonlinear model (DRNL) which was previously proposed as an active nonlinear model of the basilar membrane, the SDPN model can reproduce similar level-dependent frequency responses with a much simpler structure and is thus better suited for incorporation into CI sound processors. By the analysis of dominant frequency component, it was confirmed that the formants of speech are more robustly represented after frequency decomposition by the nonlinear filterbank using SDPN, compared to a linear bandpass filter array which is used in conventional strategies. Acoustic simulation and hearing experiments in subjects with normal hearing showed that the proposed strategy results in better syllable recognition under speech-shaped noise compared to the conventional strategy based on fixed linear bandpass filters.

## 1. Introduction

Cochlear implants (CIs) have been used successfully for the restoration of hearing function in cases of profound sensorineural hearing loss by stimulation of spiral ganglia using electrical pulses. The parameters of the electrical pulses are determined from incoming sound via sound processing strategy. Despite the great progress over a period of more than two decades, many issues remain to be resolved to achieve successful restoration of hearing in noisy environments, melody recognition, and reduction of cognitive load in the patients [1]. Hearing in a noisy environment is especially important for practical purposes.

Several methods can be utilized for the improvement of CI. Among them, the development of novel sound processing strategies is particularly useful because it can be accomplished by modifying embedded programs in the speech processor and does not require a change of hardware. A sound-processing strategy is defined here as an algorithm to generate electrical stimulation pulses based on the processing of incoming sound waveforms and is also called an encoding strategy. More accurate imitation of normal auditory function is a promising approach for CI sound-processing strategy development [1–3].

It has been suggested that speech perception performance can be improved considerably by adopting an active nonlinear model of the basilar membrane in the cochlea, called the dual resonance nonlinear (DRNL) model [2, 3]. The use of DRNL model was shown to be beneficial for the representation of the information of the formants, which mean the resonances in the vocal tract and are reflected in speech spectra as spectral peaks [2, 3]. The formants are known to be encoded in population responses of the auditory nerves [4, 5]. They are very important cues for speech perception, since the information on formants is crucial for the representation of vowels. It is also imperative for consonant representation, as formant transition provides a valuable piece of information for the identification of consonants, such as plosives, stops, and fricatives [6].

The aforementioned CI performance improvement by the use of active nonlinear model of the basilar membrane may result from robust representation of formants under noisy conditions. The DRNL model was first applied to a CI sound processor and improved speech perception performance was verified from one listener [2]. It was also reported that the DRNL-based sound-processing strategy provides robust formant representation characteristics and enhances vowel perception [3]. The DRNL model was originally developed for quantitative description of the physiological properties of the basilar membrane and to provide a satisfactory fit to experimental results. Thus, the DRNL model includes many parameters that should be determined from experimental data, and its structure is rather complicated for adoption in CI devices. Therefore, a simpler model may be implemented without compromising the advantages of the DRNL model.

Here, we propose a new active nonlinear model of the frequency response of the basilar membrane, called the simple dual path nonlinear (SDPN) model and a novel sound-processing strategy based on this model. The aim of the present study is only to utilize the advantages of the active nonlinear response and not to replicate the physiological properties of the basilar membrane in biological cochlea in detail. A subset of results has been presented in a conference proceeding [7].

## 2. Methods

### 2.1. Proposed Sound-Processing Strategy

**Figure 1:**
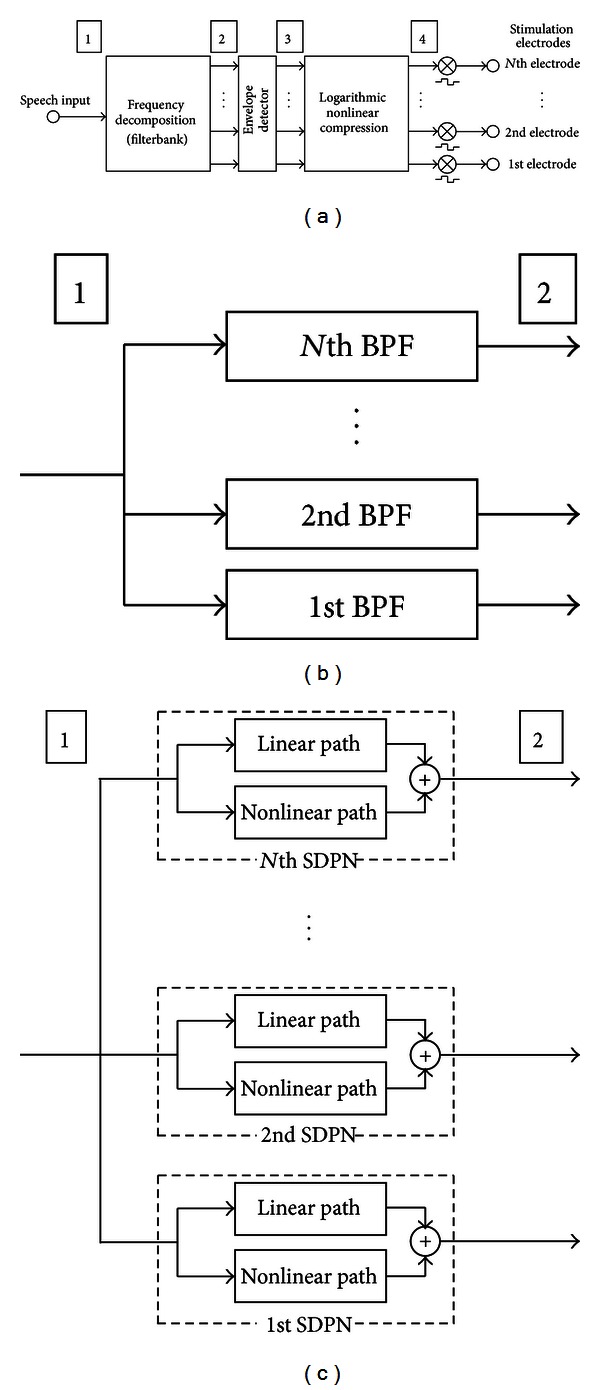
(a) General structure of CI sound-processing strategies. Incoming sound is decomposed into multiple frequency bands, and the relative strength of each subband is then determined with an envelope detector to modulate the amplitudes of stimulus pulses after logarithmic compression. (b) The frequency decomposition stage for the conventional strategy based on a fixed linear bandpass filter array. (c) The frequency decomposition stage for the proposed strategy based on the SDPN model.

Figure 1(a) shows the general structure of the sound processor for a CI. The incoming sound is decomposed into multiple frequency bands (stage 2 in Figure 1(a)), and then the relative strength of each subband is obtained from an envelope detector (stage 3) to modulate the amplitudes of stimulus pulses after logarithmic compression. This structure was motivated by place coding (tonotopy) of the basilar membrane and most modern CI devices are based on this structure [8–10]. In the strategy proposed in this paper, the frequency decomposition stage is replaced with a simple active nonlinear filter model of the basilar membrane with variable response instead of a fixed linear bandpass filter which is employed in conventional CIs. The variable response characteristic originates from the input-dependent tuning property of the basilar membrane resulting from active motility of outer hair cells (OHC) [11] and this active nonlinear response property contributes to robust representation of speech cues under noisy conditions [12].

Figures 1(b) and 1(c) illustrate the differences between the conventional and proposed strategies. Both can be regarded as having the structure shown in Figure 1(a). In the conventional strategy (Figure 1(b)), a fixed linear bandpass filter array, is adopted as the frequency decomposition block of Figure 1(a). In contrast, in the proposed strategy (Figure 1(c)), frequency decomposition is performed by the SDPN model array. The output from each channel can be regarded as a bandpass-filtered version of the input, similarly to the conventional strategy. However, the frequency response property is nonlinear and level dependent. Subsequently, the relative strength of each channel is calculated by applying envelope detectors to the outputs from each SDPN. The envelopes are used to modulate the amplitudes of the current pulses in clinical applications involving electrical stimulation; for acoustic simulation, the amplitudes of sinusoids are modulated instead of pulse amplitudes. This is described later in detail (Section 3.4).

**Figure 2:**
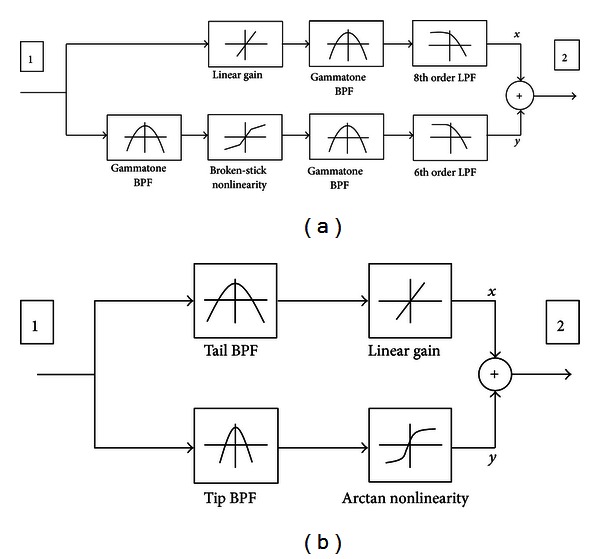
(a) Block diagram of the DRNL model. The output of each cochlear partition is represented as a summation of the outputs from a linear and a nonlinear pathway. (b) Block diagram of the proposed SDPN model.

Figure 2(a) illustrates the dual resonance nonlinear (DRNL) model which was developed for quantitative description of the physiological properties of the basilar membrane and to provide a satisfactory fit to experimental results [12]. The output of each cochlear partition is represented as a summation of the outputs from linear and nonlinear pathways in the DRNL model. The linear pathway consists of a linear gain, a gammatone bandpass filter, and a Butterworth lowpass filter. The nonlinear path includes broken-stick nonlinearity between two bandpass filters so that its contribution to the total output is determined by the input signal level. The details of the DRNL model and parameters were reported in [12]. The effective center frequencies of the linear and nonlinear pathways are slightly different. The relative contributions of the two pathways are variable because of the nonlinear gain in the nonlinear pathway, and therefore the overall response characteristics such as gain and bandwidth are also variable. The DRNL model can replicate the frequency response of biological cochlea in that the level-dependent tuning and level-dependent gain properties could be reproduced successfully [12]. Compared to other models with similar purposes, it is relatively simple and computationally efficient. However, the DRNL model includes many parameters and its structure is rather too complicated for adoption in CI devices.

**Figure 3:**
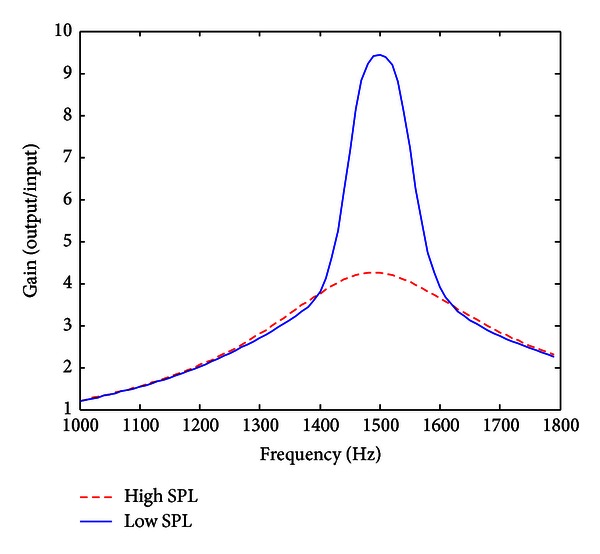
The frequency response of the proposed SDPN model when the center frequency is set to 1500 Hz. When the input amplitude is low, the contribution of the nonlinear pathway is relatively large so that the overall response shows a sharp frequency selectivity determined by the tip filter. As the amplitude increases, the contribution of linear pathway becomes dominant, and the overall frequency response therefore becomes broader.

The block diagram of the SDPN model is shown in Figure 2(b). While developing the SDPN model, we did not attempt to reproduce experimental results regarding the neurophysiological properties of the basilar membranes to the numerical details.The purpose here was to implement the level-dependent frequency response characteristics of the biological cochlea. As in the DRNL model, the incoming sound is passed to two pathways. The linear pathway consists of a linear gain (fixed to 6 here) and a broad bandpass filter, which is called the tail filter. The nonlinear pathway is made of a sharper bandpass filter, which is called the tip filter, and a compressive nonlinearity that is employed to mimic the saturation properties of the OHC. The nonlinearity is expressed as *y* = 2 arctan⁡(15*x*). Both the tail and tip filters are composed of Butterworth bandpass filters (tail filter: 2nd order, tip filter: 4th order). The bandwidth of the tail filter is set to be three times larger than that of the tip filter. To realize the variable response properties, the relative contribution of each pathway is controlled according to the input level (root mean square value) by the nonlinearity. The overall output from one channel of the frequency decomposition block is obtained by summing the outputs from the two pathways. As discussed later in Section 3 (Figure 3), this method allows the implementation of active nonlinear frequency response characteristics of biological cochlea with much lower computational costs than the DRNL model.

After frequency decomposition, the envelopes of each channel output are obtained. We used a conventional envelope detector consisting of a rectifier and a low-pass filter. In addition, we also examined the advantages of using an enhanced envelope detector proposed by Geurts and Wouters [13]. This is based on the adaptation effect resulting from the synapse between inner hair cells and auditory nerves and utilizes a combination of two envelope detectors, namely, a standard envelope detector consisting of a full-wave rectifier and a 4th order Butterworth low-pass filter with 400-Hz cutoff frequency and another for extraction of slowly varying envelope with a low-pass filter cutoff frequency of 20 Hz. By comparing the two envelopes, it is possible to determine the temporal points where rapid transient changes occur, and additional gain can be applied at these time points for emphasis of the transients. The detailed algorithm was reported in [13].

### 2.2. Acoustic Simulation

Acoustic simulation can be used to predict performance trends of CI sound-processing strategies and has therefore been utilized for many studies of the development of novel strategies [14]. We adopted sinusoidal modulation for the synthesis of acoustic waveforms, as in many previous studies on CI sound-processing strategy development [14, 15]. The center frequencies of the channels were chosen according to the method of Loizou et al. [16], as this enables systematic computation of the filter bandwidths and is used in current CI devices. Logarithmic filter spacing was used for 4-channel implementation, and semilogarithmic mel spacing was used for 8 and 12 channels. Detailed values of the center frequencies and bandwidths are listed in Table 1.

The method of acoustic simulation in the conventional strategy was similar to that of Dorman et al. [17]. After frequency decomposition of incoming sound by a linear bandpass filter array, an envelope detector consisting of a full-wave rectifier and a 4th order Butterworth low-pass filter (cutoff frequency: 400 Hz) was applied. The detected envelopes were used to modulate the sinusoids with frequencies the same as the center frequencies listed in Table 1. Finally, the amplitude-modulated sinusoids from all the channels were summed.

For the generation of an acoustic waveform corresponding to the proposed strategy, frequency decomposition was performed by an array of SDPN models, and then the envelopes of the outputs from each SDPN model were extracted by envelope detectors. Either conventional or enhanced envelope detectors were adopted. The amplitudes of sinusoids were modulated according to the outputs from the envelope detectors. The frequencies of sinusoids were the same as in the simulation using the conventional strategy. Note that we assigned one sinusoid per channel, as the center frequencies of the tail and tip filters were identical. Thus, the results of acoustic simulation can be readily compared to those of the conventional strategy. This is different from the case of acoustic simulation of the DRNL-based sound-processing strategy [2, 3], where two sinusoids should be used to simulate one channel due to the different center frequencies of linear and nonlinear pathways.

### 2.3. Hearing Experiment

Ten subjects with normal hearing volunteered to participate in the hearing experiment (mean ± SD age: 25.8 ± 4.08 years; 6 men, 4 women). All subjects were undergraduate or graduate students of Yonsei University. The experimental procedure was reviewed and approved by a local ethics review committee. The experiments were performed under two noise conditions: without any noise (i.e., signal-to-noise ratio (SNR) of *∞* dB) and with speech-shaped noise (SSN) of 2 dB SNR. The SSN here was generated by applying a 2nd order Butterworth low-pass filter (cutoff frequency 1100 Hz) to white Gaussian noise (WGN) as described previously [18] so that its spectral shape was similar to that of speech waveforms. The number of channels was varied to 4, 8, or 12 channels.

Syllable identification tests were performed using closed-set tasks. Consonant-vowel-consonant-vowel (CVCV) disyllables were constructed mainly to test vowel perception performance. Each speech token was fixed to the form of /sVda/; that is, only the first vowel was changed whereas the others were fixed to /s/, /d/, and /a/. The first vowel was selected from /a/, /*ǝ*/, /o/, /u/, /i/, and /e/. This CVCV form is more natural for the Korean language and was therefore used instead of the CVC-type monosyllables frequently utilized in vowel perception tests in previous studies [13, 17]. Vowel-consonant-vowel (VCV) type monosyllables were also constructed. The vowels at the beginning and end were the same and fixed to /a/. The consonants between vowels were selected from /g/, /b/, /m/, /n/, /s/, and /j/. Thus, the speech materials were of the /aCa/ type. A total of 72-/sVda-/ type disyllables and 72-/aCa-/ type monosyllables were generated (72 = 6 consonants/vowels × 2 strategies (conventional/SDPN-based) × 2 noise levels × 3 channel types). Two experimental sessions were performed with the same subjects; the first compared conventional and SDPN-based strategies, and the second compared the conventional strategy with that based on the SDPN and the enhanced envelope detector.

The acoustic waveforms of speech tokens were generated by 16-bit mono analog-to-digital conversion at sampling rate of 22.050 kHz and stored as  .wav files. The stored files were played by clicking icons displayed in a graphical user interface on a personal computer prepared for the experimental run. The speech tokens were presented binaurally using headphones (Sennheiser HD25SP1) and a 16-bit sound card (SoundMAX integrated digital audio soundcard). The sound level was controlled to be comfortable for each subject (range: ~70–80 dB). A 5 min training session was given before the main experiment. Each speech token was presented once. The conditions of sound processing strategies and noise conditions were randomized across subjects. If the subjects requested, the waveforms were played once more. After hearing each speech token, the subjects were instructed to choose the presented syllable among six given examples as correctly as possible, and the percentage of correct answers was scored.

## 3. Results

### 3.1. Variable Frequency Response of the SDPN Model

Figure 3 shows the frequency response of the proposed SDPN model with a center frequency of 1500 Hz. When the input amplitude was low (35 dB sound pressure level (SPL)), the contribution of the nonlinear pathway was relatively large, and so the overall response showed sharp frequency selectivity determined by the tip filter. Peak gain was 9.44, and the full width at half maximum (FWHM) was 140.27 Hz. As the amplitude increased (85 dB SPL), the contribution of the linear pathway became dominant, and the overall frequency response became broader (FWHM = 424.08 Hz). Meanwhile, the overall gain decreased due to the compressive nonlinearity (peak gain = 4.26). Overall, the frequency response of the SDPN model showed level-dependent behavior, which was similar to that of the biological cochlea. Compared to the DRNL model, the proposed simplified structure could be executed very quickly. For example, to process 1 s of sound, the CPU time was 0.054 ± 0.012 s (mean ± SD) for the SDPN model, whereas that for the DRNL was 1.33 ± 0.034 s (average of 40 trials, Matlab implementation, 3.0 GHz Pentium 4 processor, 2 GB RAM). That is, the processing time for the proposed SDPN model was only about 1/24.6 that of the DRNL model.

### 3.2. Formant Representation under Noisy Conditions

**Figure 4:**
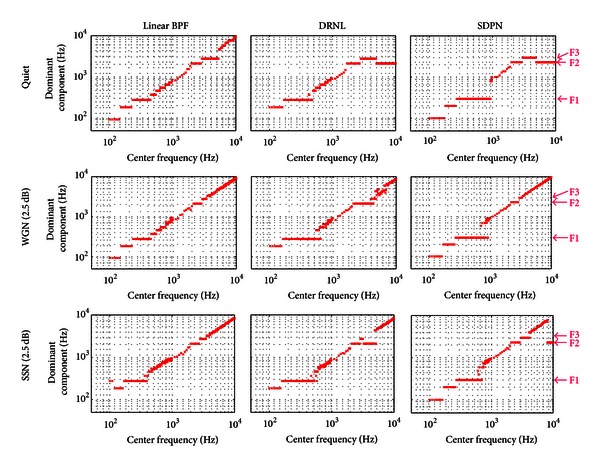
Dominant frequency component analysis for the vowel /i/. F1, F2, and F3 are at 270 Hz, 2290 Hz, and 3010 Hz, respectively. Upper row: under quiet conditions. Middle row: under 2.5 dB WGN. Lower row: under 2.5 dB SSN. Left column: by the linear BPF array. Middle columns: DRNL. Right column: SDPN.

The superiority of the active nonlinear models for robust representation of formants under noisy conditions could be demonstrated by dominant frequency component analysis, that is, by plotting the maximum frequencies of the output from each cochlear partition as a function of the center frequency [19]. We divided the frequency range from 100 Hz to 10 kHz in 181 partitions and observed the output from each cochlear partition.  Figure 4 shows the results of dominant frequency component analysis after frequency decomposition using the fixed linear bandpass filter, the DRNL model, and the proposed SDPN model (input: vowel /i/, under quiet conditions, 5 dB WGN, and 5 dB SSN). Particularly under noisy conditions, the maximum frequencies of the outputs from active nonlinear models (DRNL and SDPN) were concentrated at the location of formant frequencies, as shown by the horizontal lines at the formants, whereas those from the linear filterbank model were determined by the center frequencies of each channel so that the data points were more concentrated at diagonal locations. Thus, the proposed SDPN model is more effective for robust formant representation under noisy conditions than the linear filter array and has advantages similar to those of the DRNL model. Similar results were also obtained for /a/ and /u/. 

**Figure 5:**
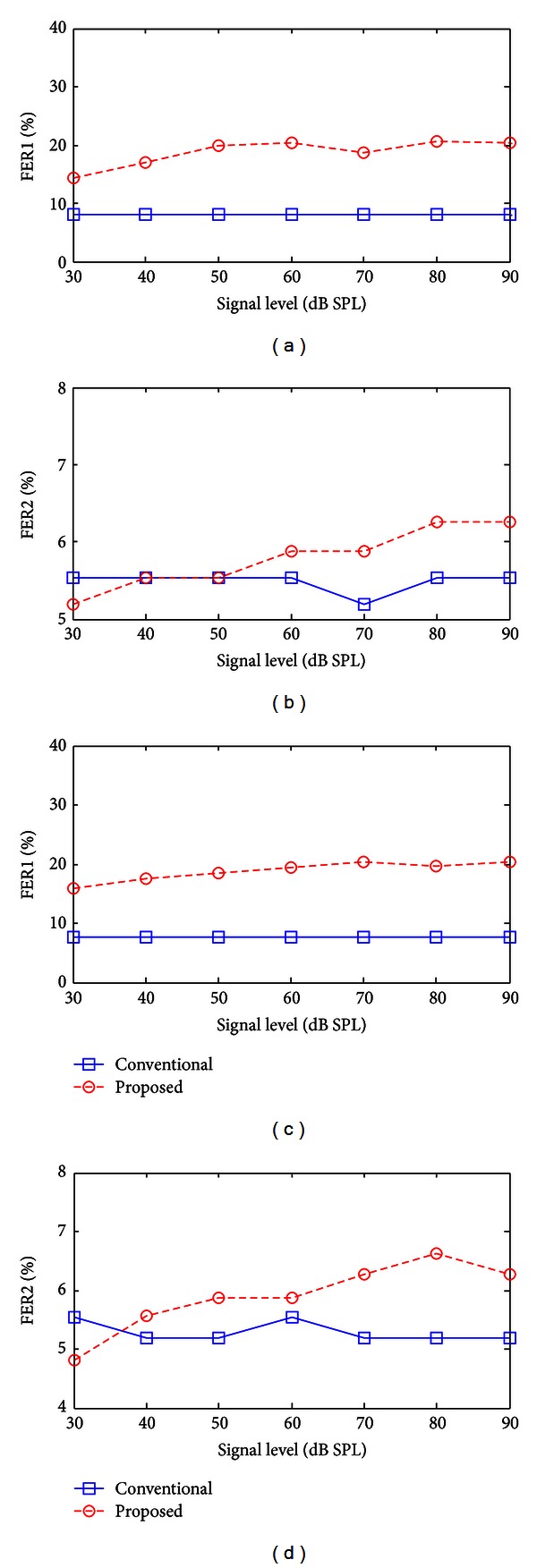
FER1 ((a) and (c)) and FER2 ((b) and (d)) at various sound pressure levels (SPLs) for the vowel /i/. (a) and (b) under WGN of 2.5 dB SNR. (c) and (d) under SSN of 2.5 dB SNR.

From the results of dominant frequency component analysis, formant representation performance could be quantified by counting the number of cochlear partitions the maximum output frequencies of which were determined by the formant frequencies. We defined two formant extraction ratios (FERs), FER1 and FER2, as the ratios of cochlear partitions with maximum output frequencies that were the same as the 1st and 2nd formant frequencies, respectively. FER1 and FER2 can be regarded as good quantitative measures of saliency of the formant representation in the output speech. Since the performance of nonlinear models could vary according to the input level as the response characteristic changes with respect to the input level, we observed the changes in formant representation performance at various SPLs.  Figure 5 shows FER1 and FER2 for the vowel /i/ as functions of input amplitude under conditions of WGN and SSN of 5 dB SNR. For a wide range of input levels, the SDPN yielded higher FER1 and FER2 compared to the linear bandpass filter under both WGN and SSN. The FERs of the linear model remained constant except for slight fluctuations due to error. As shown in Figures 5(a) and 5(b), the SDPN resulted in higher values of FER1 at all input amplitudes under WGN. The FER2 of the SDPN was also higher than that of the linear model when the SPL was higher than 40 dB. This indicated that the SDPN is advantageous for the formant representation for typical SPL levels. The SDPN was also superior when the SSN was added as background noise (Figures 5(b) and 5(d)).

### 3.3. Enhanced Envelope Detector

**Figure 6:**
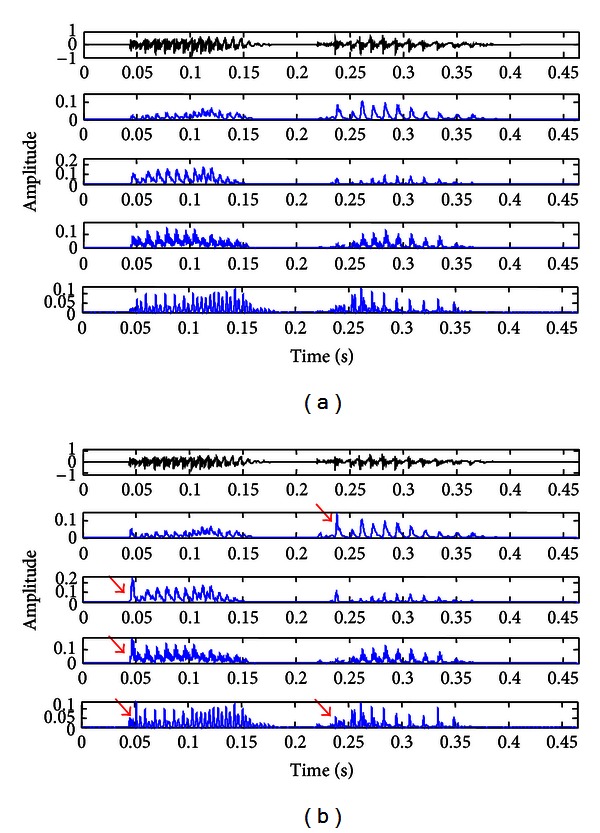
The envelopes obtained from (a) conventional and (b) enhanced envelope detectors after frequency decomposition by the SDPN model. The arrows in (b) indicate emphasis of speech onset.

Figure 6 shows the envelopes of 4 channels obtained from conventional (Figure 6(a)) and enhanced (Figure 6(b)) envelope detectors after frequency decomposition using the SDPN model. The arrows in Figure 6(b) indicate the time points where the enhanced envelope detector effectively emphasized the point of speech onset. Particularly, for the input speech “/aka/,” the onset point of /k/ was significantly accentuated in Figure 6(b).

### 3.4. Acoustic Simulation and Hearing Experiment

**Figure 7:**
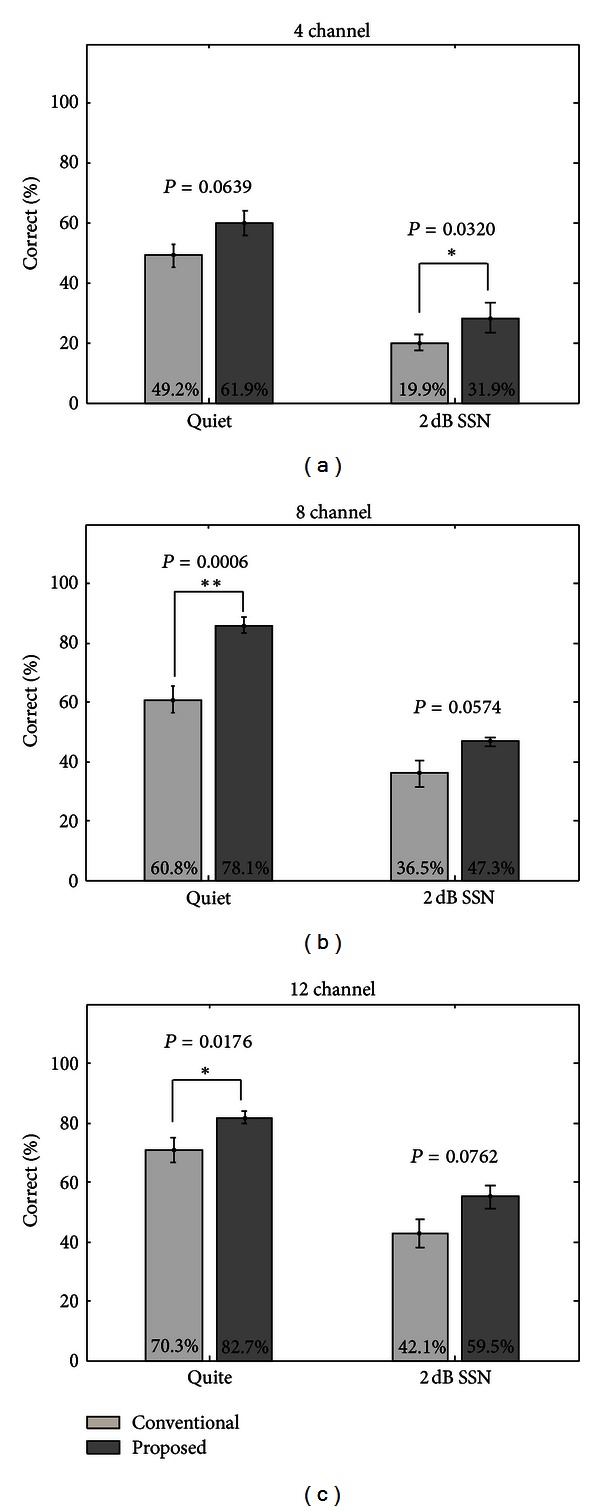
Results of syllable identification tests using the sound-processing strategy based on the SDPN and the conventional envelope detector (under quiet conditions or SSN of 2 dB SSN). (a) 4 channels. (b) 8 channels. (c) 12 channels.

**Figure 8:**
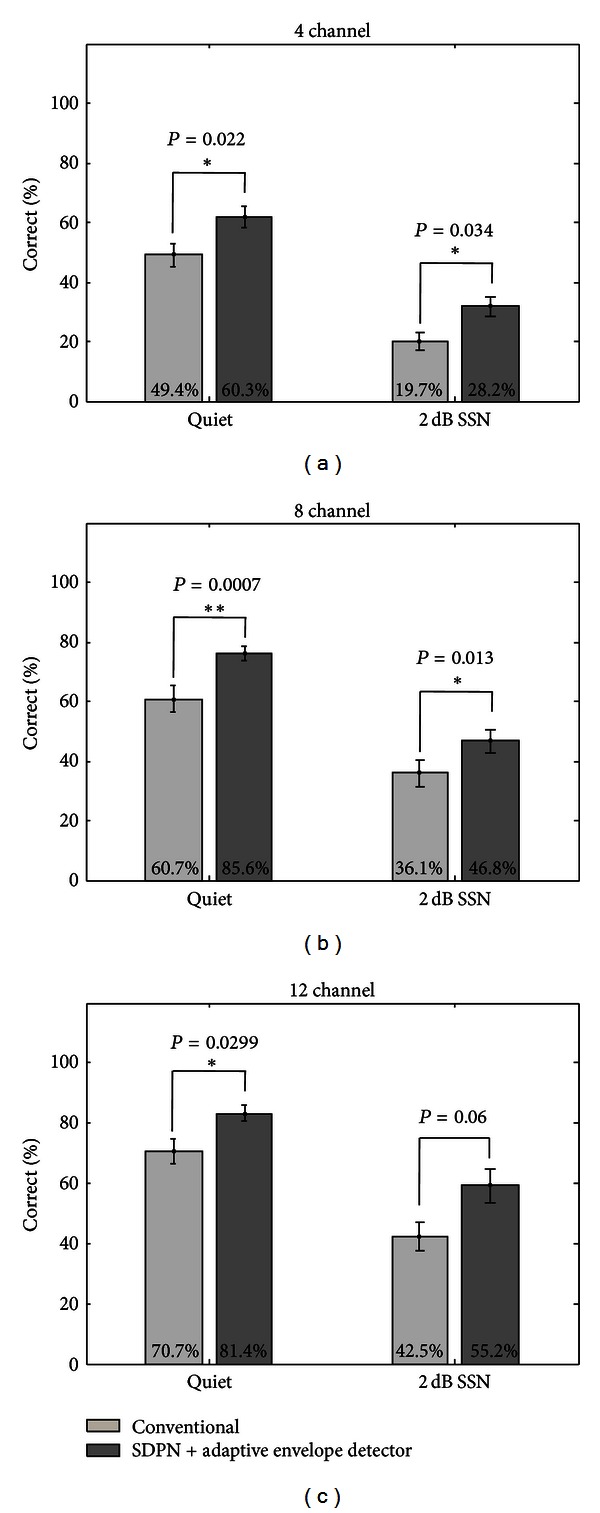
Results of syllable identification tests using the sound-processing strategy based on the SDPN and the enhanced envelope detector (under quiet conditions or SSN of 2 dB SSN). (a) 4 channels. (b) 8 channels. (c) 12 channels.

The results of hearing experiments using acoustic simulation of the proposed sound-processing strategy based on the SDPN model are shown in Figure 7. The percentages of correct answers were plotted as functions of the number of channels for 4, 8, and 12 channels. For all conditions, the proposed strategy was considerably superior to the conventional strategy. Although statistical significance (*P* < 0.05) was not reached for some conditions, the proposed strategy yielded much better speech perception performance for all conditions; all *P*-values were <0.0762 and approached statistical significance.  Figure 8 shows the results of hearing experiments using a strategy based on the SDPN and the enhanced envelope detector. For quiet conditions, the proposed strategy was better than the conventional one for all channel conditions. The superiority was statistically significant for all channel conditions (*t*-test, *P* < 0.05 for 4 channels, and *P* < 0.01 for 8 and 12 channels). Under SSN of 2 dB SNR, the proposed strategy provided considerably better syllable identification for all channel conditions (*t*-test, *P* < 0.05 for 4 and 8 channels, *P* = 0.06 for 12 channels).

## 4. Discussion

In this study, we proposed a simple active nonlinear model of basilar membrane in the cochlea and developed a novel sound-processing strategy for the CIs based on this model. Acoustic simulation and hearing experiments in subjects with normal hearing indicated that the proposed strategy provides enhanced syllable identification performance under conditions of speech-shaped noise, compared to the conventional strategy using a fixed linear bandpass filter array.

Some previous experimental studies indicated that the active nonlinear frequency response property contributes significantly to robust representation of formant information in noisy environments. Several models were suggested to reproduce this property [11, 20, 21]. For example, Deng and Geisler [11] proposed a nonlinear differential equation model with a variable damping term to simulate a level-dependent compression effect and successfully reconstructed the response characteristics of the biological cochlea that are beneficial for robust spectral cue representation under noise. This implies that the speech perception performance of CIs can be improved by adopting the active nonlinear response property, as demonstrated by the enhanced performance of CI sound-processing strategy based on the DRNL model [2, 3].

Although the DRNL model is one of the most efficient models in terms of computational costs, its purposes are to quantitative description of the physiological properties of the basilar membrane and to replicate detailed experimental results. The complicated structure and numerous parameters of the DRNL model make it unsuitable for the CI sound processor. The motivation for development of the SDPN model was to simplify the DRNL model without compromising its advantages due to the adaptive nonlinear frequency response. The SDPN model was developed as a further simplification of the DRNL model, with the purpose of developing a CI sound-processing strategy. The emphasis was on reproducing the input-dependent response characteristics of biological cochlea qualitatively. Many building blocks and parameters of the DRNL model were not necessary to implement the level-dependent frequency response of the biological cochlea, because they were adopted for the detailed replication of experimental results and are not essential to our goal here. The proposed SDPN is much simpler than the DRNL but can still provide the level-dependent frequency response, which is beneficial for real-time processing with lower power consumption due to less computation. 

The results of dominant frequency analysis verified that more robust formant representation under SSN could be obtained from the proposed SDPN model. When the SDPN model was used, the output frequency was dominated by formant frequencies in much more cochlear partitions compared to the case of the linear bandpass filterbank (Figures 4 and 5). Despite the simplification, the formant representation performance of the SDPN model was comparable to that of the DRNL presented in [3], as can be verified by the results of dominant frequency component analysis and FERs. This suggests that the detailed imitation of the frequency response characteristics of the human basilar membrane is not essential for the improvement of CI speech perception performance. This is in contrast with a previous study [2] in which a detailed model of human basilar membrane based on the DRNL model was adopted in the CI sound processor. 

The comparison between the envelopes extracted by two envelope detectors shown in Figure 6 showed that the enhanced envelope detector provides the emphasis of speech onset points, which is often weak in amplitude. This property may contribute to the improvement of the perception of stop, fricative, and plosive consonants. This was confirmed from the hearing experiments using acoustic simulation (Figures 7 and 8), as the use of the enhanced envelope detector provided further improvement of the SDPN-based strategy in speech perception.

A new sound-processing strategy for CI should be applied in clinical tests for more comprehensive verification. This requires the modulation of electrical pulse trains based on the sound processor output. The proposed SDPN-based strategy was developed so that it employs one amplitude-modulated pulse train per channel in actual CI devices. Thus, it is readily applicable to the existing hardware of current CIs.

In conclusion, we proposed a simple novel model of active nonlinear characteristics of biological cochlea and developed a sound-processing strategy for CI based on the model. The proposed SDPN model was based on the function of the basilar membrane so that a level-dependent frequency response can be reproduced; it is much simpler than the DRNL model and is thus better suited for incorporation into CI sound processors. The SDPN-based strategy was evaluated by spectral analysis and hearing experiments in subjects with normal hearing. The results indicated that the use of the SDPN model provides advantages similar to those of the DRNL-based strategy in that the formant is more robustly represented under noisy conditions. Further improvement in speech perception under noisy conditions was possible by adopting an enhanced envelope detector.

## Figures and Tables

**Table 1a:** (a) 4 Channel implementation

	Ch. 1	Ch. 2	Ch. 3	Ch. 4
	CFs and BWs of BPFs (in conventional strategy)			

CF (Hz)	460	953	1971	4078
BW (Hz)	321	664	1373	2426

	CFs and BWs of tip and tail BPFs (in proposed strategy)			

CF (Hz)	460	953	1971	4078
BW of tip filter (Hz)	321	664	1373	2426
BW of tail filter (Hz)	107	221.3	457.7	808.7

**Table 1b:** (b) 8 Channel implementation

	Ch. 1	Ch. 2	Ch. 3	Ch. 4	Ch. 5	Ch. 6	Ch. 7	Ch. 8
	CFs and BWs of BPFs (in conventional strategy)							

CF (Hz)	394	692	1064	1528	2109	2834	3740	4871
BW (Hz)	265	331	431	516	645	805	1006	1257

	CFs and BWs of tip and tail BPFs (in proposed strategy)							

CF (Hz)	394	692	1064	1528	2109	2834	3740	4871
BW of tip filter (Hz)	265	331	431	516	645	805	1006	1257
BW of tail filter (Hz)	83.3	110.3	143.7	172	215	268.3	335.3	419

**Table 1c:** (c) 12 Channel implementation

	Ch. 1	Ch. 2	Ch. 3	Ch. 4	Ch. 5	Ch. 6	Ch. 7	Ch. 8	Ch. 9	Ch. 10	Ch. 11	Ch. 12
	CFs and BWs of BPFs (in conventional strategy)											

CF (Hz)	274	453	662	905	1190	1521	1908	2359	2885	3499	4215	5050
BW (Hz)	165	193	225	262	306	357	416	486	567	661	771	900

	CFs and BWs of tip and tail BPFs (in proposed strategy)											

CF (Hz)	274	453	662	905	1190	1521	1908	2359	2885	3499	4215	5050
BW of tip filter (Hz)	165	193	225	262	306	357	416	486	567	661	771	900
BW of tail filter (Hz)	55	64.3	75	87.3	102	119	138.7	162	189	220.3	257	300

CF: center frequency, BPF: bandpass filter, BW: bandwidth.

## References

[1] Wilson BS, Lawson DT, Muller JM, Tyler RS, Kiefer J (2003). Cochlear implants: some likely next steps. *Annual Review of Biomedical Engineering*.

[2] Schatzer R, Wilson BS, Wolford RD, Lawson DT (2003). Speech processors for auditory prostheses: signal processing strategy for a closer mimicking of normal auditory functions. *Sixth Quatery Progress Report*.

[3] Kim KH, Choi SJ, Kim JH, Kim DH (2009). An improved speech processing strategy for cochlear implants based on an active nonlinear filterbank model of the biological cochlea. *IEEE Transactions on Biomedical Engineering*.

[4] Palmer AR, Winter IM, Darwin CJ (1986). The representation of steady-state vowel sounds in the temporal discharge patterns of the guinea pig cochlear nerve and primarylike cochlear nucleus neurons. *Journal of the Acoustical Society of America*.

[5] Bandyopadhyay S, Young ED (2004). Discrimination of voiced stop consonants based on auditory nerve discharges. *Journal of Neuroscience*.

[6] Young ED, Sachs MB (1979). Representation of steady-state vowels in the temporal aspects of the discharge patterns of populations of auditory-nerve fibers. *Journal of the Acoustical Society of America*.

[7] Kim KH, Choi SJ, Kim JH A speech processing strategy for cochlear implant based on a simple dual path nonlinear model of Basilar membrane.

[8] Wilson B, Finley C (1991). Improved speech recognition with cochlear implants. *Nature*.

[9] Loizou P (1999). Signal-processing techniques for cochlear implants. *IEEE Engineering in Medicine and Biology Magazine*.

[10] Rubinstein JT (2004). How cochlear implants encode speech. *Current Opinion in Otolaryngology & Head and Neck Surgery*.

[11] Deng L, Geisler CD (1987). A composite auditory model for processing speech sounds. *Journal of the Acoustical Society of America*.

[12] Meddis R, O’Mard LP, Lopez-Poveda EA (2001). A computational algorithm for computing nonlinear auditory frequency selectivity. *Journal of the Acoustical Society of America*.

[13] Geurts BL, Wouters J (1999). Enhancing the speech envelope of continuous interleaved sampling processors for cochlear implants. *Journal of the Acoustical Society of America*.

[14] Dorman MF, Spahr AJ, Loizou PC, Dana CJ, Schmidt JS (2005). Acoustic simulations of combined electric and acoustic hearing (EAS). *Ear and Hearing*.

[15] Zeng FG, Nie K, Stickney GS (2005). Speech recognition with amplitude and frequency modulations. *Proceedings of the National Academy of Sciences of the United States of America*.

[16] Loizou PC, Dorman M, Tu Z (1999). On the number of channels needed to understand speech. *Journal of the Acoustical Society of America*.

[17] Dorman MF, Loizou PC, Rainey D (1997). Speech intelligibility as a function of the number of channels of stimulation for signal processors using sine-wave and noise-band outputs. *Journal of the Acoustical Society of America*.

[18] Yang LP, Fu QJ (2005). Spectral subtraction-based speech enhancement for cochlear implant patients in background noise. *Journal of the Acoustical Society of America*.

[19] Holmes SD, Sumner CJ, O’Mard LP, Meddis R (2004). The temporal representation of speech in a nonlinear model of the guinea pig cochlea. *Journal of the Acoustical Society of America*.

[20] Robert A, Eriksson JL (1999). A composite model of the auditory periphery for simulating responses to complex sounds. *Journal of the Acoustical Society of America*.

[21] Tan Q, Carney LH (2003). A phenomenological model for the responses of auditory-nerve fibers. II. Nonlinear tuning with a frequency glide. *Journal of the Acoustical Society of America*.

